# A circular RNA blood panel that differentiates Alzheimer’s disease from other dementia types

**DOI:** 10.1186/s40364-022-00405-0

**Published:** 2022-08-18

**Authors:** Ziye Ren, Changbiao Chu, Yana Pang, Huimin Cai, Longfei Jia

**Affiliations:** Innovation Center for Neurological Disorders and Department of Neurology, Xuanwu Hospital, Capital Medical University, National Clinical Research Center for Geriatric Diseases, 45 Changchun St, Beijing, China

**Keywords:** Alzheimer’s disease, Dementia, circRNA, Biomarker, Diagnosis

## Abstract

**Background:**

Circular RNAs (circRNAs) have been demonstrated to be associated with Alzheimer’s disease (AD). Here, we conducted a study to explore whether circRNAs have the ability to differentiate AD from cognitively normal controls and other types of dementia, such as vascular dementia (VaD), Parkinson’s disease dementia (PDD), behavioral variant frontotemporal dementia (bvFTD), and dementia with Lewy body (DLB).

**Methods:**

Three datasets were included in this study to measure blood circRNAs. The pilot study (Dataset 1, *n* = 40; controls, 20; AD, 20) was used to screen differentially expressed circRNAs. Dataset 2 (*n* = 124; controls, 61; AD, 63) was recruited for the establishment of the diagnostic model using a circRNA panel. Further, the Dataset 3 (*n* = 321; control, 58; AD, 60; VaD, 50; PDD, 51; bvFTD, 52; DLB, 50) was used to verify the diagnostic model.

**Results:**

In Dataset 1, 22 upregulated and 19 downregulated circRNAs were revealed. In Dataset 2, a six-circRNA panel was found to be able to distinguish patients with AD from controls. Then this panel was applied to Dataset 3 and successfully differentiated AD from other types of dementia.

**Conclusion:**

This study suggested that a six-circRNA panel is AD-specific and a promising biomarker of AD.

**Supplementary Information:**

The online version contains supplementary material available at 10.1186/s40364-022-00405-0.

## Background

Alzheimer’s disease (AD) is the most common neurodegenerative disease-causing dementia. AD affects the quality of life of patients and imposes a heavy burden on their families and society [1]. Currently, diagnosis is mainly assisted by neuroimaging or cerebrospinal fluid (CSF) biomarkers, which can reflect amyloid beta-protein (Aβ) deposition and neuronal damage in the brain [2]. However, these examinations are expensive, invasive, and time-consuming. In addition, the differential diagnosis of AD is difficult. There are similar clinical manifestations, pathologies, and biomarkers between AD and other dementias such as vascular dementia (VaD), Parkinson’s disease dementia (PDD), behavioral variant frontotemporal dementia (bvFTD), and dementia with Lewy body (DLB) [3]. Therefore, it is necessary to identify peripheral biomarkers to distinguish AD from other types of dementia.

Circular RNAs (circRNAs) are a class of non-coding RNA molecules with closed loops [4]. CircRNAs play a biological role by sponging miRNAs, regulating intracellular RNA-binding proteins, and participating in protein translation [5]. Recent studies have found that circRNAs are highly expressed in the nervous system, particularly in the brain [6]. They are involved in signal transduction in the nervous system and the regulation of various neural activities [7]. In terms of stability, specificity and tissue abundance, circRNAs are considered to be promising biomarkers for monitoring AD. Studies have found that there are differentially expressed circRNAs in the brain tissue and plasma of AD patients [8–10]. However, the results of previous studies have been inconsistent [11, 12]. One reason for this could be the small sample size of the studies. In the study by Li et al., there were only five patients with AD. The small sample size of the studies may have caused differences in the results. Another possible reason for the inconsistencies is the inclusion criteria, which did not include CSF or positron emission tomography (PET) biomarkers to recruit patients with AD. This study used CSF biomarkers to recruit patients with AD, and the sample size was relatively large, increasing the accuracy of the results.

In this study, we recruited participants for three independent datasets and aimed to evaluate whether the levels of blood circRNAs (1) can be used to differentiate patients with AD from cognitively normal controls and (2) can effectively discriminate AD from VaD, PDD, bvFTD, and DLB.

## Materials and methods

### Experimental design

For the three sets of data, a total of 485 subjects were included in this study (Tables 1, 2 and 3, Additional file 1: Fig. S1). Dataset 1 participants were recruited from a Beijing center for the pilot study (*n* = 40; controls, 20; AD, 20); Dataset 2 participants were recruited from centers in the provinces of Shandong, Henan, and Guangxi for the development of the diagnostic model (*n* = 124; control, 61; AD, 63); Dataset 3 participants were recruited from the Beijing Center for the validation of the model (*n* = 321; control, 58; AD, 60; VaD, 50; PDD, 51; bvFTD, 52; DLB, 50). The diagnosis of AD was based on the criteria of the National Institute on Aging and Alzheimer’s Association (NIA-AA) [13]. We additionally used a cutoff value for the phosphorylated tau (P-tau)/Aβ42 > 0.14 in CSF to distinguish patients with AD from normal controls, which was confirmed in our previously published data [14, 15]. Based on the Amyloid/Tau/Neurodegeneration (ATN) framework, low Aβ42 levels in CSF are considered to be the key pathological changes in AD [16]. Consequently, the CSF Aβ42 level of 500 pg/mL was included as another inclusion criterion [17]. VaD [18], PDD [19], bvFTD [20], and DLB [21] were diagnosed according to the previously published criteria. However, other dementias may have overlapping clinical manifestations, pathologies, and biomarkers with AD, making the clinical diagnosis difficult. To avoid confounding AD with other dementias, patients with VaD, PDD, bvFTD, and DLB who met the cutoff values for P-tau/Aβ42 and Aβ42 in AD were excluded. The Institutional Ethics Board of Xuanwu Hospital, Capital Medical University, approved this study, and written informed consent was obtained from all participants or their legal representatives before enrollment.

**Table 1:** Characteristics of participants in dataset 1

Characteristic	Total Sample (*n* = 40)	Controls (*n* = 20)	AD (*n* = 20)
Age, mean (SD)	69.6 (5.5)	69.7 (6.5)	69.4 (4.5)
Education year, mean (SD)	9.3 (2.2)	9 (2.1)	9.6 (2.4)
Women, No. (%)	20 (50.0)	10 (50.0)	10 (50.0)
*ApoE* ε4 positive (%)	12 (30.0)	4 (20.0)	8 (40.0)^*^
MMSE score, mean (SD)	24.3 (5.3)	28.8 (0.5)	19.8 (3.8)^*^
Aβ42, mean (SD), pg/ml	568.7 (219.1)	736.5 (189.1)	400.9 (59)^*^
T-tau, mean (SD), pg/ml	486.9 (206.5)	315.6 (56.7)	658.1 (150.2)^*^
P-tau, mean (SD), pg/ml	96 (51.3)	62.3 (34.5)	129.7 (42.5)^*^

The values of age, education year, MMSE, Aβ42, T-tau, and P-tau are shown as mean (SD)

Abbreviations: *AD* Alzheimer’s disease, *VaD* vascular dementia, *PDD* Parkinson disease dementia, *bvFTD* behavioral variant frontotemporal dementia, *DLB* dementia with Lewy body, *ApoE* ε4 apolipoprotein ε4, *MMSE* Mini-Mental State Examination, *SD* standard deviation

^*^P < 0.05 compared to controls

**Table 2:** Characteristics of participants in dataset 2

Characteristic	Total Sample (*n* = 124)	Controls (*n* = 61)	AD (*n* = 63)
Age, mean (SD)	69.2 (6.8)	68.9 (6.9)	69.5 (6.8)
Education year, mean (SD)	9.4 (2.3)	9.6 (2.0)	9.2 (2.5)
Women, No. (%)	63 (50.8)	31 (50.8)	32 (50.8)
*ApoE* ε4 positive (%)	37 (29.8)	11 (18.0)	26 (41.3)^*^
MMSE score, mean (SD)	24.8 (4.6)	29 (0.6)	20.8 (2.9)^*^
Aβ42, mean (SD), pg/ml	543 (217.1)	723.1 (152.1)	368.5 (90.7)^*^
T-tau, mean (SD), pg/ml	478.1 (198.3)	331.0 (97.7)	620.5 (163.9)^*^
P-tau, mean (SD), pg/ml	89.3 (59.3)	52 (30.6)	125.4 (58.1)^*^

The values of age, education year, MMSE, Aβ42, T-tau, and P-tau are shown as mean (SD)

Abbreviations: *AD* Alzheimer’s disease, *VaD* vascular dementia, *PDD* Parkinson disease dementia, *bvFTD* behavioral variant frontotemporal dementia, *DLB* dementia with Lewy body, *ApoE* ε4 apolipoprotein ε4, *MMSE* Mini-Mental State Examination, *SD* standard deviation

^*^P < 0.05 compared to controls

**Table 3:** Characteristics of participants in dataset 3

Characteristic	Total Sample (*n* = 321)	Controls (*n* = 58)	AD (*n* = 60)	VaD (*n* = 50)	PDD (*n* = 51)	bvFTD (*n* = 52)	DLB (*n* = 50)
Age, mean (SD)	68.6 (6.7)	68.8 (7.5)	67.6 (6.8)	69.5 (5.9)	69.6 (7.3)	67.8 (6.9)	68.4 (5.6)
Education year, mean (SD)	9.4 (2.2)	9.1 (1.8)	9.1 (2.2)	9.2 (2.2)	9.4 (2.4)	9.6 (2.2)	10.1 (2.3)
Women, No. (%)	165 (51.4)	29 (50.0)	31 (51.7)	26 (52.0)	26 (51.0)	27 (51.9)	26 (52.0)
*ApoE* ε4 positive (%)	77 (24.0)	10 (17.2)	25 (41.7)^*^	12 (24.0)	10 (19.6)	10 (19.2)	10 (20)
MMSE score, mean (SD)	22 (4.2)	29.1 (0.6)	19.6 (2.6)^*^	19.9 (2.8)^*^	21.4 (2.6)^*^	19.7 (2.7)^*^	21.6 (2.9)^*^
Aβ42, mean (SD), pg/ml	631.7 (193)	703.8 (123.5)	346.6 (81.6)^*^	713.5 (123.6)	741.6 (162.4)	706.3 (125.4)	655.1 (120.4)
T-tau, mean (SD), pg/ml	423.8 (148.1)	343.9 (136.9)	596.2 (145.8)^*^	412.2 (102.6)	369.9 (100.9)	409.9 (110.9)	390.6 (116.8)
P-tau, mean (SD), pg/ml	59.2 (33.5)	52 (23.4)	104.6 (49)^*^	46.9 (12.9)	50.3 (15.4)	48.2 (11)	46 (12)

The values of age, education year, MMSE, Aβ42, T-tau, and P-tau are shown as mean (SD)

Abbreviations: *AD* Alzheimer’s disease, *VaD* vascular dementia, *PDD* Parkinson disease dementia, *bvFTD* behavioral variant frontotemporal dementia, *DLB* dementia with Lewy body, *ApoE* ε4 apolipoprotein ε4, *MMSE* Mini-Mental State Examination, *SD* standard deviation

^*^P < 0.05 compared to controls

### RNA collection and sequencing

The participants fasted for 12 h before blood samples were collected in the morning. We collected 20 mL of whole blood in a polypropylene tube containing EDTA. Whole blood samples were processed immediately at the Beijing Center. At the other centers, the collected samples were preliminarily processed to obtain the plasma. Samples were centrifuged at 4200×*g* at room temperature for 10 min, the plasma was collected, kept at 4 °C, and delivered to the Beijing Central Laboratory within 12 h. Total RNA was isolated using the miRNeasy Serum Kit (Qiagen, USA) according to the manufacturer’s instructions. One microgram of total RNA was used to prepare the sequencing library (quantified with a Nano Drop 8000 [Thermo Fisher Scientific, USA] and Agilent 2100 bioanalyzer [Agilent, USA]). An additional 3 μg of total RNA was treated with DNase I to degrade double- and single-stranded DNA. To further purify poly-A RNA, we used the Ribo-off rRNA Depletion Kit (Vazyme, Inc.) to deplete ribosomal RNA and RNase R (New England Biolabs Inc., USA) to remove linear RNA. Agencourt RNAClean XP magnetic beads were used for purification. All procedures were performed according to the manufacturer’s protocol. Two methods were used to qualify the library: the Agilent 2100 bioanalyzer was used to test the distribution of the fragment size, and BMG (OMEGA) was used to quantify the library. Finally, BGISEQ-500 (BGI-Shenzhen, China) was used to sequence the pair ends for the qualified libraries.

### CircRNA data analysis

To filter the sequencing data, we applied SOAPnuke (v1.5.2) in the following three ways [22]: 1) removing reads that contain sequencing adapters; 2) removing reads with a low-quality base ratio (base quality ≤5) greater than 20%; and 3) removing reads with an unknown base (‘N’ base) ratio greater than 5%. As a result, we obtained clean reads and stored them in the FASTQ format. HISAT2 (v2.0.4) was then applied to map the clean reads to the reference genome [23]. Subsequently, fusion genes and differential splicing genes were detected using Ericscript (v0.5.5) [24] and rMATS (V3.2.5) [25]. We used Bowtie2 (v2.2.5) [26] to align the clean reads with the gene set built by the Beijing Genomic Institute in Shenzhen, which is a database that includes known and novel coding and noncoding transcripts. Next, we calculated the expression levels of the genes using RSEM (v1.2.12) [27]. Differential expression analysis was performed using DESeq2 (v1.4.5) [28] with a Q value of ≤0.05.

### Collection of CSF and measurement of Aβ42, T-tau, and P-tau

CSF samples were collected immediately after blood collection, following international guidelines [29]. Specifically, the subject was positioned in the left lateral position, and a lumbar puncture was performed to collect 15 mL of CSF. CSF samples were centrifuged at 2000×*g* for 10 min at room temperature and stored in polypropylene tubes at − 80 °C. Enzyme-linked immunosorbent assay (ELISA) kits were used to measure the levels of Aβ42, total tau (T-tau), and P-tau181 in the CSF. (Additional file 1: Table S1).

### Statistical analysis

SPSS v.22 and Stata 13.0 were used to perform the statistical analysis, and the three datasets were analyzed independently. Categorical data between groups, such as sex and apolipoprotein E (APOE) ε4 distributions, were compared using the χ2 test. Continuous data between groups, such as biomarker concentrations, were compared using Welch’s *t*-test or analysis of variance (ANOVAs). To select the differentially expressed circRNAs in Dataset 1, we used the false discovery rate (FDR) to correct *P-*values and showed the analysis results with Q values. Then, the predicted values were generated in Datasets 2 and 3 using a binary logistic regression model based on covariates of age, sex, education years, and APOE ε4 state, which was subsequently used for receiver operating characteristic (ROC) curve analysis. Multicollinearity between each circRNA was calculated using tolerances, variance inflation factors (VIFs), eigenvalues, and condition indices [30]. All tests were two-tailed, and statistical significance was set at *P* < 0.05.

## Results

### Participant characteristics

The characteristics of the participants in the three datasets are presented in Tables 1, 2 and 3. In the three sets of data, the AD and control groups showed no differences in age or the male-to-female ratio. In Datasets 1 and 2, there were statistically significant differences (*P* < 0.05) between AD patients and controls in APOE ε4 percentage, and results of the Mini-Mental State Examination (MMSE). Compared to the control group, the MMSE scores for VaD, PDD, bvFTD, and DLB in Dataset 3 were also reduced (all *P* < 0.05).

### Pilot study

**Fig. 1:**
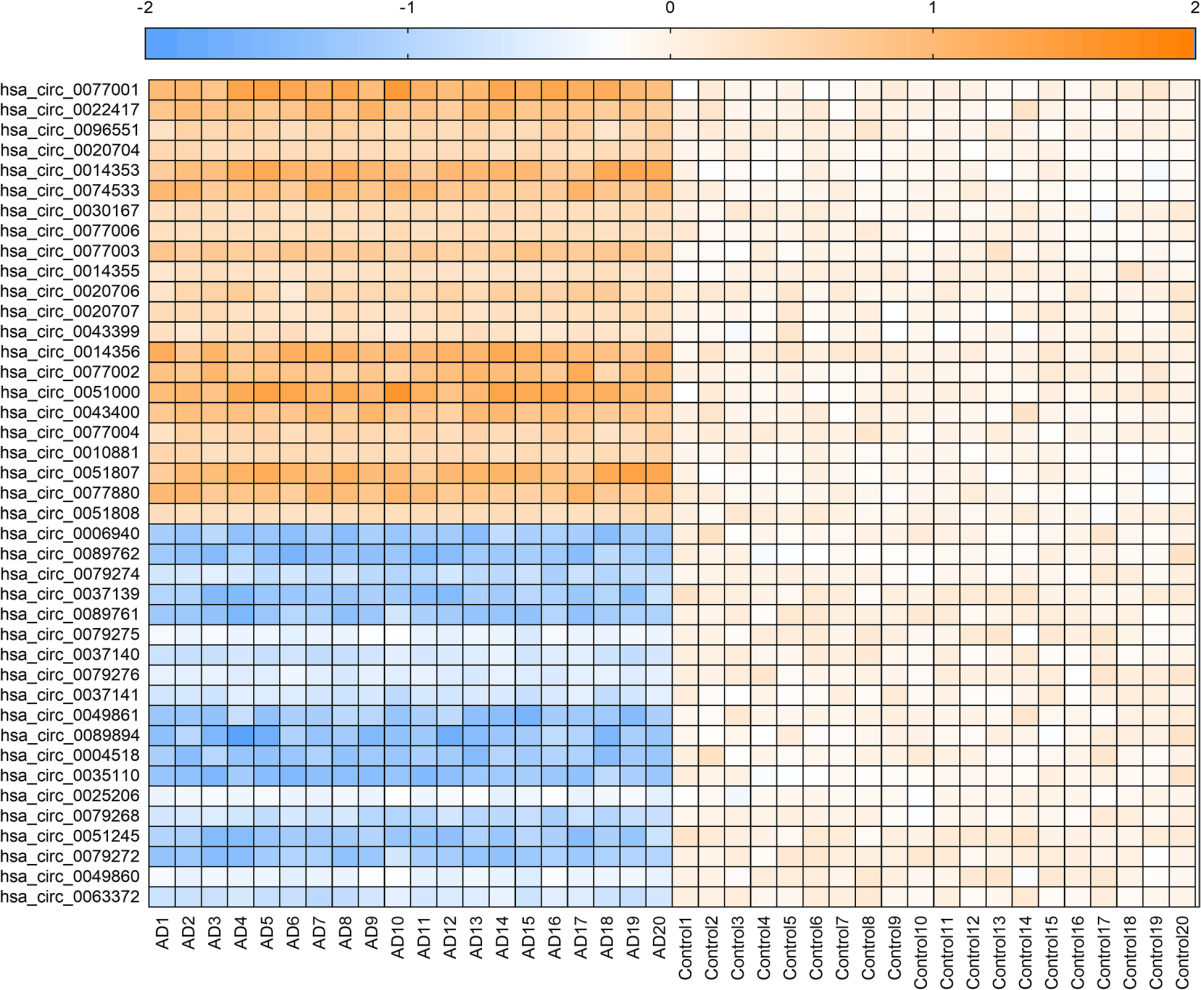
Heat map of 22 upregulated and 19 downregulated circRNAs in the pilot study (all Q < 0.05, with FDR correction, Dataset 1). Orange and blue indicate upregulation and downregulation, respectively. Abbreviations: FDR, false discovery rate; AD, Alzheimer’s disease

A pilot study was conducted using a comparatively small sample group (Dataset 1) to identify altered circRNAs. The RNA-sequencing results showed that there were 1875 circRNAs in the blood of patients with AD and the control group, whose read counts exceeded 100. CircRNAs with read counts less than 100 were excluded from the analysis. We identified 22 upregulated and 19 downregulated circRNAs in the AD group (Fig. 1, Additional file 1: Fig. S2) according to fold changes of ≥1.2 or ≤ 0.80 compared with controls.

### Establishment of the predictive model

**Fig. 2:**
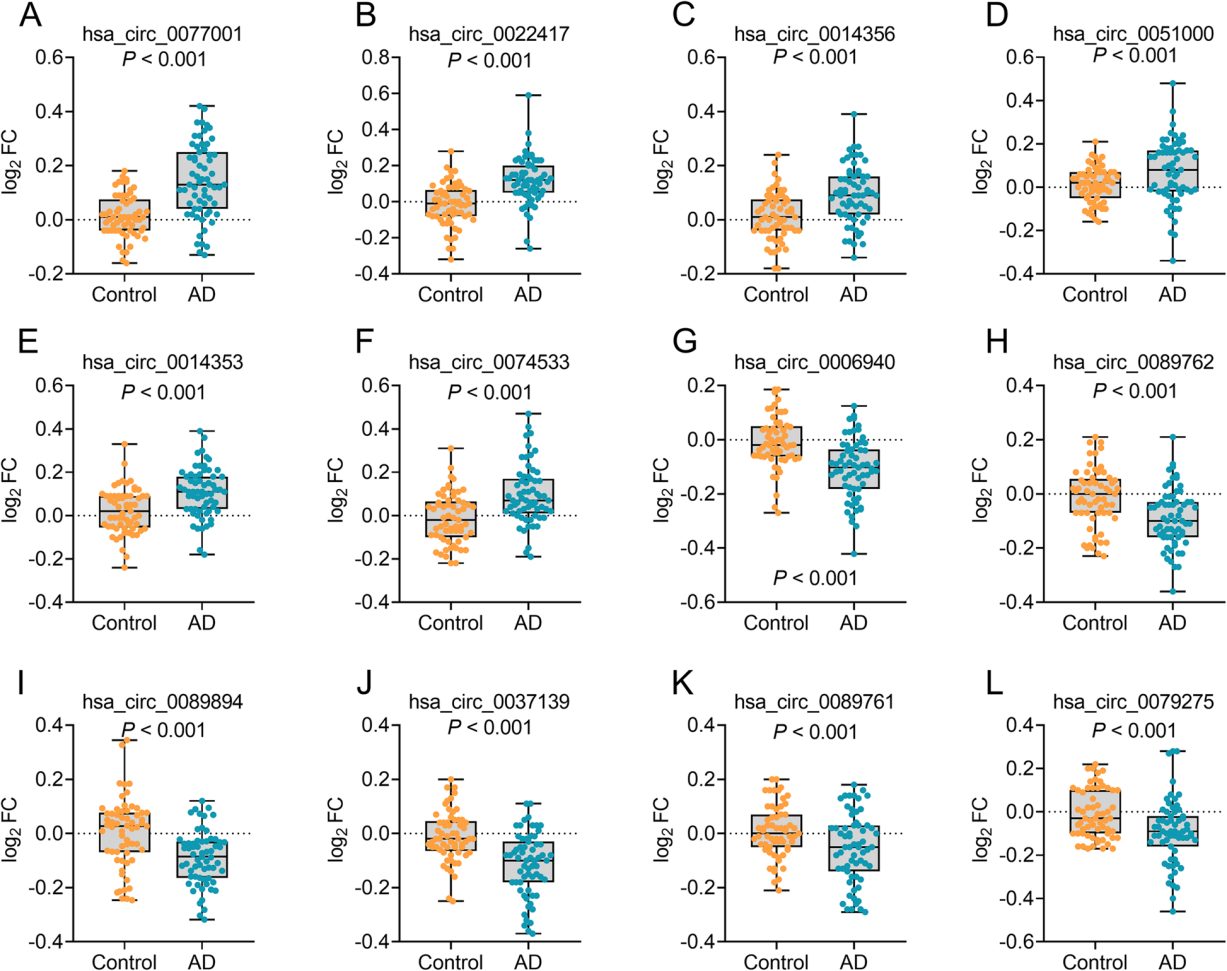
Measurements of circRNAs in Dataset 2. hsa_circ_0077001 (**A**), hsa_circ_0022417 (**B**), hsa_circ_0014356 (**C**), hsa_circ_0051000 (**D**), hsa_circ_0014353 (**E**), and hsa_circ_0074533 (**F**) were increased in patients with AD, while hsa_circ_0006940 (**G**), hsa_circ_0089762 (**H**), hsa_circ_0089894 (**I**), hsa_circ_0037139 (**J**), hsa_circ_0089761 (**K**), and hsa_circ_0079275 (**L**) were decreased in AD. Abbreviations: AD, Alzheimer’s disease; FC, fold change

**Fig. 3:**
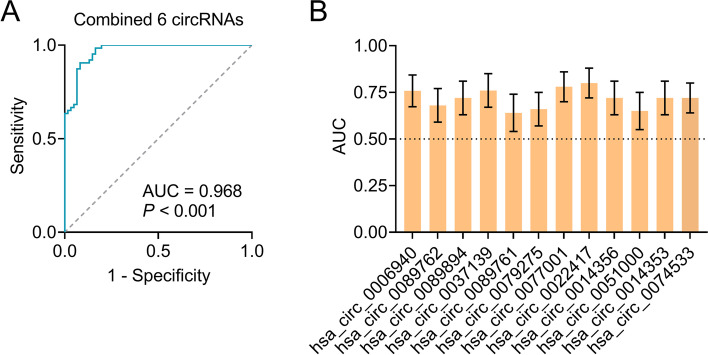
Establishment of diagnostic panel for AD. (**A**) ROC curve analysis of the six-circRNA panel (upregulated: hsa_circ_0077001, hsa_circ_0022417, hsa_circ_0014356, hsa_circ_0014353, hsa_circ_0074533; downregulated: hsa_circ_0089894). (**B**) ROC analysis of 12 individual circRNAs. Abbreviations: AD, Alzheimer’s disease; ROC, receiver operating characteristic; AUC, area under the curve

Dataset 2, with extended samples, was included in the development of the diagnostic model. The total 41 differential circRNAs were confirmed in Dataset 2, supporting that the sequencing data revealed in the pilot study were significant (Additional file 1, Table S2). The top six upregulated circRNAs (hsa_circ_0077001, hsa_circ_0022417, hsa_circ_0014356, hsa_circ_0051000, hsa_circ_0014353, and hsa_circ_0074533) and the top six downregulated circRNAs (hsa_circ_0006940, hsa_circ_0089762, hsa_circ_0089894, hsa_circ_0037139, hsa_circ_0089761, and hsa_circ_0079275) were selected for further analyses (Fig. 2). Using diagnosis (AD versus controls; AD as positive events, and controls as negative events) as the dependent variable and the 12 circRNAs, age, sex, years of education, and APOE ε4 status as covariates, we established a binary logistic regression model to assess the performance of the aforementioned 12 circRNAs in distinguishing patients with AD from controls. By stepwise forward regression, a panel of six circRNAs (upregulated: hsa_circ_0077001, hsa_circ_0022417, hsa_circ_0014356, hsa_circ_0014353, hsa_circ_0074533; downregulated: hsa_circ_0089894, all *P* < 0.05) entered into the diagnostic model, while other circRNAs, age, sex, years of education, and APOE ε4 status were excluded from the model (All *P* > 0.05). Gene ontology (GO) analysis revealed that the six circRNAs were involved in biological process, cellular component, and molecular function, such cellular process, metabolic process, immune system process, and synapse, which have demonstrated to be associated with AD [31, 32] (Additional file 1: Fig. S3). Kyoto Encyclopedia of Genes and Genomes (KEGG) pathway analysis showed that PI3K-Akt signaling pathway, insulin resistance, and cell growth and death, which are reportedly to be associated with AD [31, 33, 34]. (Additional file 1:Fig. S4). In addition, the six circRNAs also showed association with other chronic aging diseases, such as cancer, diabetes, infectious diseases, and neurodegenerative diseases (Additional file 1: Fig. S4). In further analysis, age, sex, and years of education were excluded, as their *P*-values in the logistic model were more than 0.05. The multicollinearity diagnostics of the six circRNAs in patients with AD and controls showed that all tolerances were > 0.1, VIFs were < 10, eigenvalues were > 0, and condition indices were < 30, demonstrating that there was no significant multicollinearity among the six circRNAs. We then evaluated the predictive values of the six-circRNA panel using the ROC curve analysis to validate the diagnostic ability of the panel. Further results showed that the area under the curve (AUC) of the six-circRNA panel (AUC = 0.968, *P* < 0.001, Fig. 3A) was significantly higher than that of a single circRNA (AUCs = 0.636–0.796, Fig. 3B), suggesting that the combination of the six circRNAs has an excellent diagnostic capacity for AD.

### Application of the prediction model

**Fig. 4:**
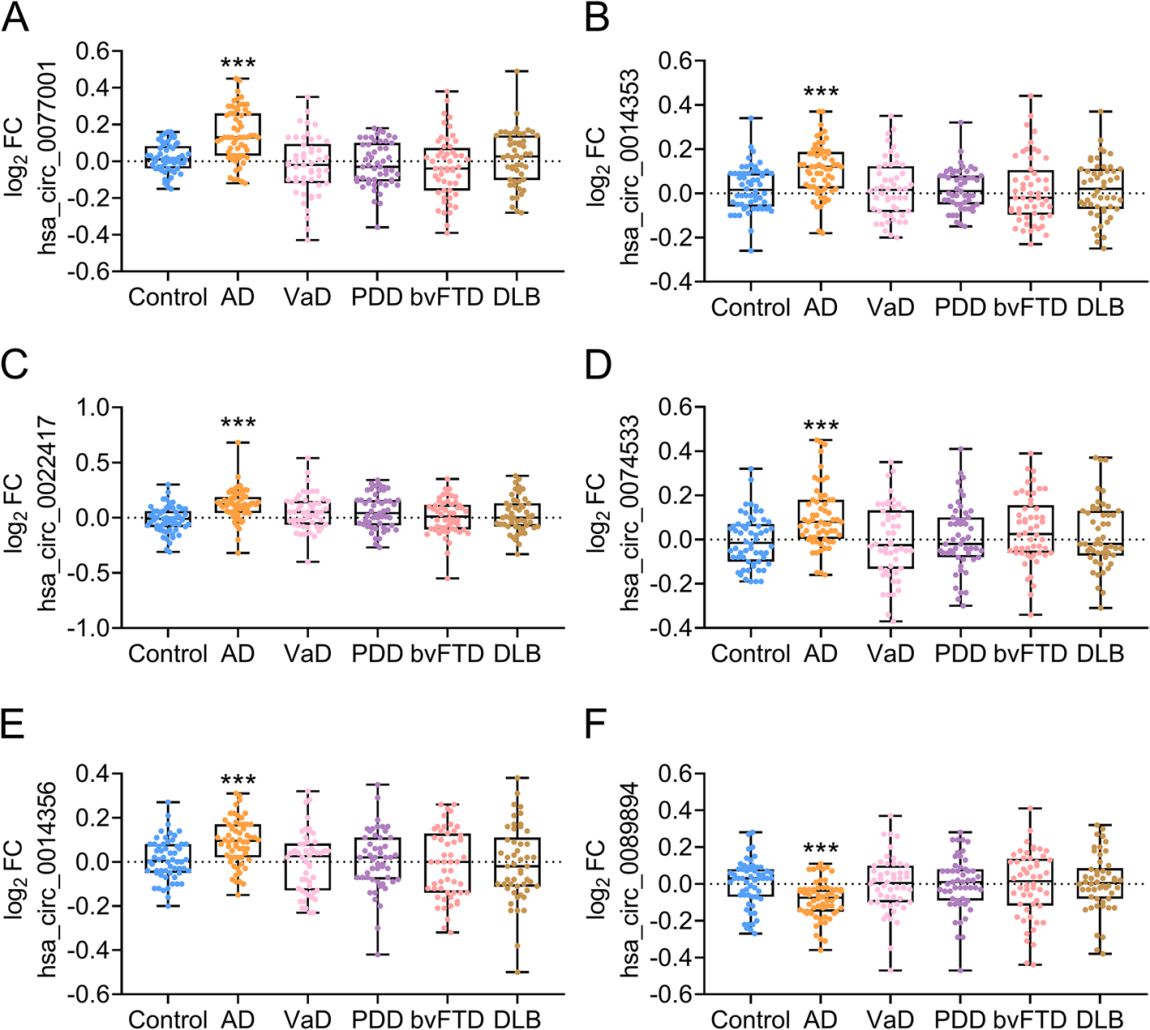
Measurements of circRNAs in control, AD, VaD, PDD, bvFTD, and DLB. hsa_circ_0077001 (**A**), hsa_circ_0014353 (**B**), hsa_circ_0022417 (**C**), hsa_circ_0074533 (**D**), hsa_circ_0014356 (**E**), and hsa_circ_0089894 (**F**) were measured. Abbreviations: AD, Alzheimer’s disease; VaD, vascular dementia; PDD, Parkinson disease dementia; bvFTD, behavioral variant frontotemporal dementia; DLB, dementia with Lewy body; FC, fold change

**Fig. 5:**
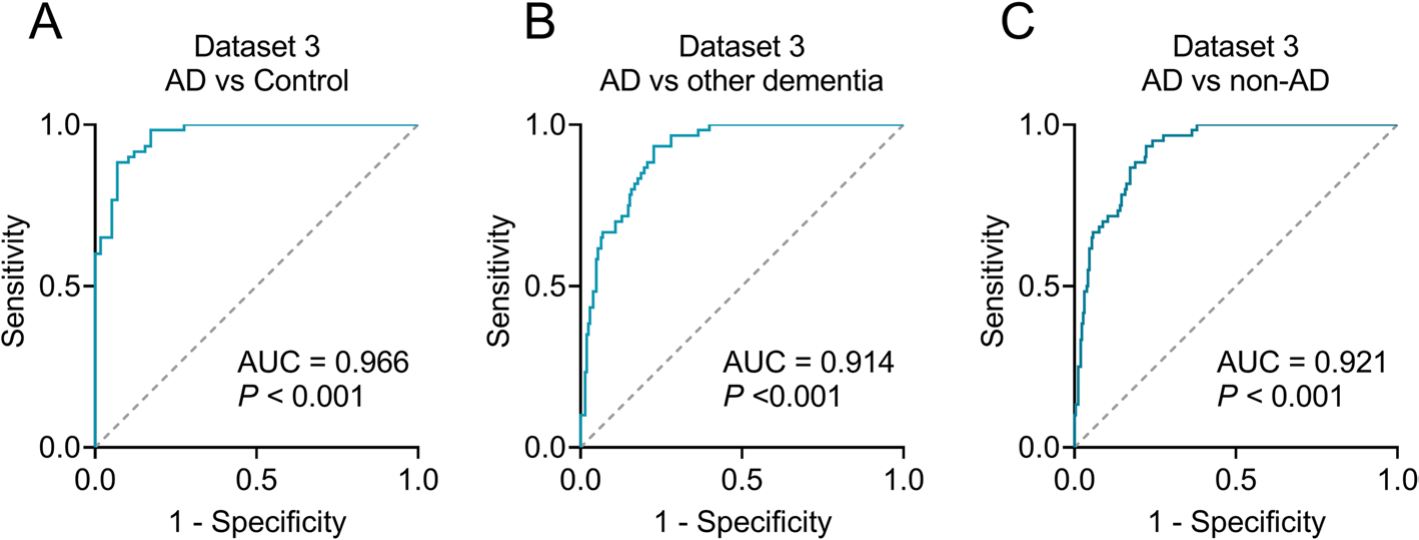
ROC curve analysis in Dataset 3. The ROCs of AD versus controls (**A**), AD versus other types of dementia (**B**), and AD versus non-AD (**C**), Non-AD indicates a combination of controls and other types of dementia. Other types of dementia include VaD, PDD, bvFTD, and DLB. Abbreviations: ROC, receiver operating characteristic; AD, Alzheimer’s disease; VaD, vascular dementia; PDD, Parkinson disease dementia; bvFTD, behavioral variant frontotemporal dementia; DLB, dementia with Lewy body; AUC, area under the curve. *** *P* < 0.001

Dataset 3 was used to estimate the differential diagnostic capacity for AD from other dementias, including VaD, PDD, bvFTD, and DLB. We obtained similar results to Datasets 1 and 2; The total 41 differential circRNAs were confirmed in Dataset 3 (Additional file 1, Table S3). In particular, the levels of hsa_circ_0077001, hsa_circ_0022417, hsa_circ_0014356, hsa_circ_0014353, and hsa_circ_0074533 were increased, whereas hsa_circ_0089894 was decreased in patients with AD (*P* < 0.001; Fig. 4A–F). None of the six circRNAs was altered in VaD, PDD, bvFTD, or DLB patients (all *P* > 0.05), indicating that these circRNAs were AD-specific. ROC analysis revealed a remarkably high AUC (0.914–0.966, *P* < 0.001, Fig. 5A–C), suggesting that the panel of six circRNAs can effectively differentiate AD from healthy controls and other dementias.

## Discussion

The present study generated a panel of circRNAs that could distinguish AD patients from controls and other types of dementia, including VaD, PDD, bvFTD, and DLB. To the best of our knowledge, this is the first effort to establish a diagnostic model of circRNAs to differentiate AD from non-AD dementia.

Biomarkers play a crucial role in AD diagnosis [13]. Decreased levels of Aβ42 and increased levels of P-tau and T-tau in the CSF are considered biomarkers of AD. However, this method is limited by its invasiveness. Therefore, the use of peripheral blood has received increasing attention. With the proliferation of research, a series of promising markers have been found in the blood, including Aβ42/40 [35], the neurofilament light protein (NFL) [36], T-tau [14], P-tau181 and 217 [37], and synaptic proteins [15, 38]. Despite their high diagnostic efficiency, the popularity of diagnostic technology is limited. The collection and measurement of existing blood markers usually require specialized skills and advanced equipment, resulting in an excessively high cost. By employing the extensively used technique of RNA-sequencing to measure a panel of blood circRNAs, our technique can differentiate AD from control and other types of dementia, and the analysis of circRNAs in the blood is antibody-independent, and minimally invasive. Taken together, our technique may be promising for AD screening in older populations.

Recent studies have increasingly implicated circRNAs in the pathology of AD [9, 39–41]. Gruner et al. observed that circRNAs in the mouse brain would accumulate during the aging process [42]. Differentially expressed circRNAs were also found in brain tissues of patients with AD [9]. Other findings suggested that altered circRNAs can alleviate the pathological manifestations of AD [40, 43]. One of the mechanisms involves sponging miRNAs to reduce their availability [44]. For example, ciRS-7 inhibits Aβ elimination by deregulating miR-7 [44]. MiR-103 is involved in axon growth and inhibits neuronal apoptosis, whereas circ_0000950 can reduce this effect by sponging [40]. By binding to mir-138-5p, circPCCA inhibits the activation of glycogen synthase kinase-3 β and promotes tau phosphorylation [10]. CircRNAs can also function by binding to proteins [45]. Chen et al. suggested that circNF1-419 can regulate inflammatory factors and the expression of marker proteins such as T-tau, P-tau, Aβ42, and APOE [39]. In addition, Ma et al. suggested that circTulp4 promotes Tulp4 transcription to regulate neuronal differentiation [41]. However, further studies on the six circRNAs in the diagnostic panel in this study are limited. The mechanisms by which these circRNAs mediate the process of development are unclear. We speculated that their regulatory roles in AD pathways in the brain make them function as AD-specific factors, which may explain why the changes in these six circRNAs can differentiate AD from other dementias.

This study had some limitations. First, the design of this study was cross-sectional, which is a disadvantage in evaluating the performance of these biomarkers. In contrast, longitudinal studies can assess the relationship between biomarker levels and cognitive decline in patients. Hence, longitudinal studies are required to analyze circRNAs. Second, this study was limited by the type of participants. To establish a diagnostic model, we recruited healthy controls, patients with AD, and other types of dementia, but lacked patients with mild cognitive impairment. This will lower the predictive ability of our method in the development of the prodromal stage to possible AD. Finally, using RNA-sequencing to measure circRNA is a quantification method that cannot reveal the absolute levels of circRNAs in the blood, making it difficult to compare the absolute levels of circRNA in our study with others.

## Conclusions

Taken together, the findings of this study suggested that the six-circRNA panel is a promising biomarker of AD. In addition, the diagnostic circRNA panel can make differential diagnosis between AD and other types of dementia, further emphasizing its potential clinical value. However, longitudinal studies are needed in further research.

## Supplementary Information

**Additional file 1: Fig. 1.** The experimental flow chart. Abbreviations: AD, Alzheimer’s disease; ROC, receiver operating characteristic; AUC, area under the curve; VaD, vascular dementia; PDD, Parkinson disease dementia; bvFTD, behavioral variant frontotemporal dementia; DLB, dementia with Lewy body. **Fig. 2.** Volcano plot of altered circRNAs in the pilot study (*P*<0.05). There are 41 circRNAs move from 1875 circRNAs according to fold changes of ≥1.2 or ≤ 0.80 compared with controls. Red and blue indicate upregulation and downregulation, respectively. Abbreviations: FC, fold change. **Fig. 3.** GO enrichment analysis of the 6 differentially expressed circRNAs. (A) The GO enrichment analysis revealed that the differentially expressed circRNAs were enriched in biological process and cellular component, molecular function. (B) Correlation network of the GO enrichment pathways. Abbreviations: GO, gene ontology. **Fig. 4.** KEGG pathway enrichment analysis of the 6 differentially expressed circRNAs. (A) The top 20 KEGG enrichment pathways. (B) Classification of KEGG enrichment pathways. (C) Correlation network of the KEGG enrichment pathways. Abbreviations: KEGG, Kyoto Encyclopedia of Genes and Genomes. **Table 1.** ELISA kits information. **Table 2.** Confirmation of differential circRNAs in Dataset 2. Abbreviations: CircRNA, Circular RNA; AD, Alzheimer’s disease; Has, Homo sapiens; FC, fold change. **Table 3.** Confirmation of differential circRNAs in Dataset 3. Abbreviations: CircRNA, circular RNA; AD, Alzheimer’s disease; Has, Homo sapiens; FC, fold change.

## Data Availability

The datasets used and/or analyzed during the current study are available from the corresponding author on reasonable request.

## Abbreviations

AD: Alzheimer’s disease
VaD: Vascular dementia
PDD: Parkinson’s disease dementia
bvFTD: Behavioral variant frontotemporal dementia
DLB: Dementia with Lewy bodies
CSF: Cerebrospinal fluid
Aβ: Amyloid beta
CircRNAs: Circular RNAs
PET: Positron emission tomography
NIA-AA: National Institute on Aging and Alzheimer’s Association
P-tau: Phosphorylated tau
ATN: Amyloid/Tau/Neurodegeneration
ELISA: Enzyme-linked immunosorbent assay
T-tau: Total tau
APOE: Apolipoprotein E
ANOVA: Analysis of variance
FDR: False discovery rate
ROC: Receiver operating characteristic
VIFs: Variance inflation factors
MMSE: Mini-Mental State Examination
GO: Gene ontology
KEGG: Kyoto Encyclopedia of Genes and Genomes
AUC: Area under the curve
NFL: Neurofilament light protein
