# Supplementary material for: A circular RNA blood panel that differentiates Alzheimer’s disease from other dementia types
Source: Biomark Res. 2022 Aug 18;10:63. doi: 10.1186/s40364-022-00405-0 (PMC9389828; doi:10.1186/s40364-022-00405-0)
Supplement: Supplementary file 1 — Additional file 1: Fig. 1. The experimental flow chart. Abbreviations: AD, Alzheimer’s disease; ROC, receiver operating characteristic; AUC, area under the curve; VaD, vascular dementia; PDD, Parkinson disease dementia; bvFTD, behavioral variant frontotemporal dementia; DLB, dementia with Lewy body. Fig. 2. Volcano plot of altered circRNAs in the pilot study (P<0.05). There are 41 circRNAs move from 1875 circRNAs according to fold changes of ≥1.2 or ≤ 0.80 compared with controls. Red and blue indicate upregulation and downregulation, respectively. Abbreviations: FC, fold change. Fig. 3. GO enrichment analysis of the 6 differentially expressed circRNAs. (A) The GO enrichment analysis revealed that the differentially expressed circRNAs were enriched in biological process and cellular component, molecular function. (B) Correlation network of the GO enrichment pathways. Abbreviations: GO, gene ontology. Fig. 4. KEGG pathway enrichment analysis of the 6 differentially expressed circRNAs. (A) The top 20 KEGG enrichment pathways. (B) Classification of KEGG enrichment pathways. (C) Correlation network of the KEGG enrichment pathways. Abbreviations: KEGG, Kyoto Encyclopedia of Genes and Genomes. Table 1. ELISA kits information. Table 2. Confirmation of differential circRNAs in Dataset 2. Abbreviations: CircRNA, Circular RNA; AD, Alzheimer’s disease; Has, Homo sapiens; FC, fold change. Table 3. Confirmation of differential circRNAs in Dataset 3. Abbreviations: CircRNA, circular RNA; AD, Alzheimer’s disease; Has, Homo sapiens; FC, fold change. [file 40364_2022_405_MOESM1_ESM.pdf]

Supplementary Material

Title:

A circular RNA blood panel that differentiates Alzheimer's disease from other dementia types

Ziye Ren[1], MD; Changbiao Chu[1], MD, PhD; Yana Pang, MS; Huimin Cai, MD; Longfei Jia*

The Supplementary materials include the following information:

1.Figures

Figure 1. The experimental flow chart.

Figure 2. Volcano plot of altered circRNAs in the pilot study ($P < 0.05$).

Figure 3. GO enrichment analysis of the 6 differentially expressed circRNAs.

Figure 4. KEGG pathway enrichment analysis of the 6 differentially expressed circRNAs.

2.Tables

Table 1. ELISA kits information

Table 2. Confirmation of differential circRNAs in Dataset 2.

Table 3. Confirmation of differential circRNAs in Dataset 3.

1.Figures

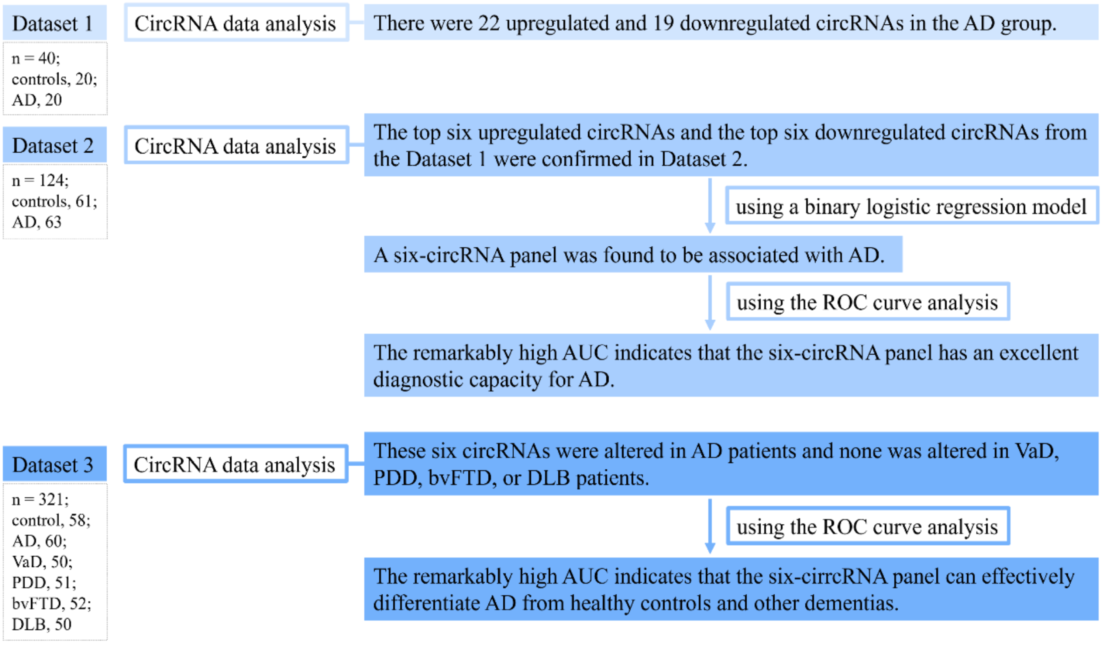

Figure 1. The experimental flow chart. Abbreviations: AD, Alzheimer's disease; ROC, receiver operating characteristic; AUC, area under the curve; VaD, vascular dementia; PDD, Parkinson disease dementia; bvFTD, behavioral variant frontotemporal dementia; DLB, dementia with Lewy body.

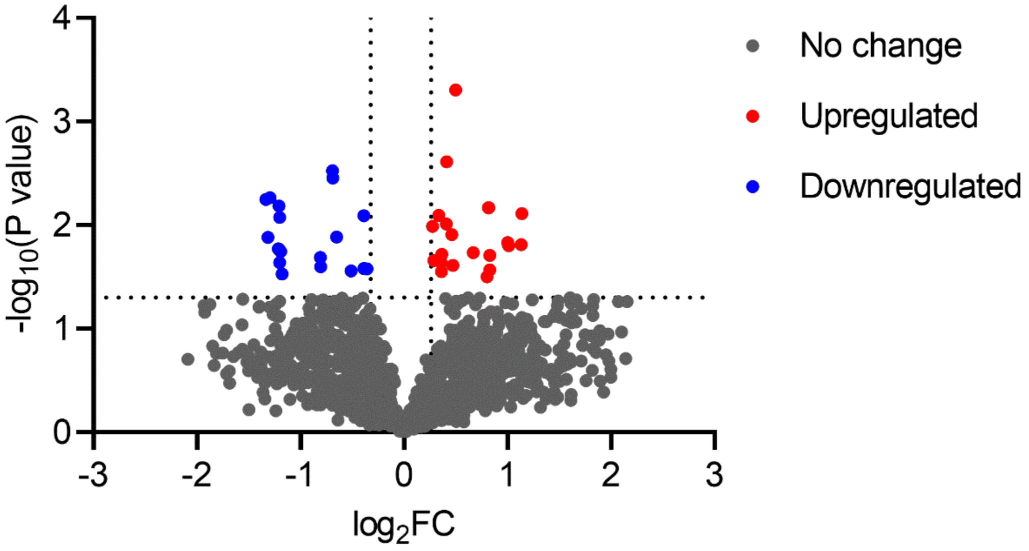

Figure 2. Volcano plot of altered circRNAs in the pilot study. There were 41 circRNAs that significantly upregulated or downregulated in AD among 1875

circRNAs. Red and blue indicate upregulation and downregulation, respectively. Abbreviations: FC, fold change.

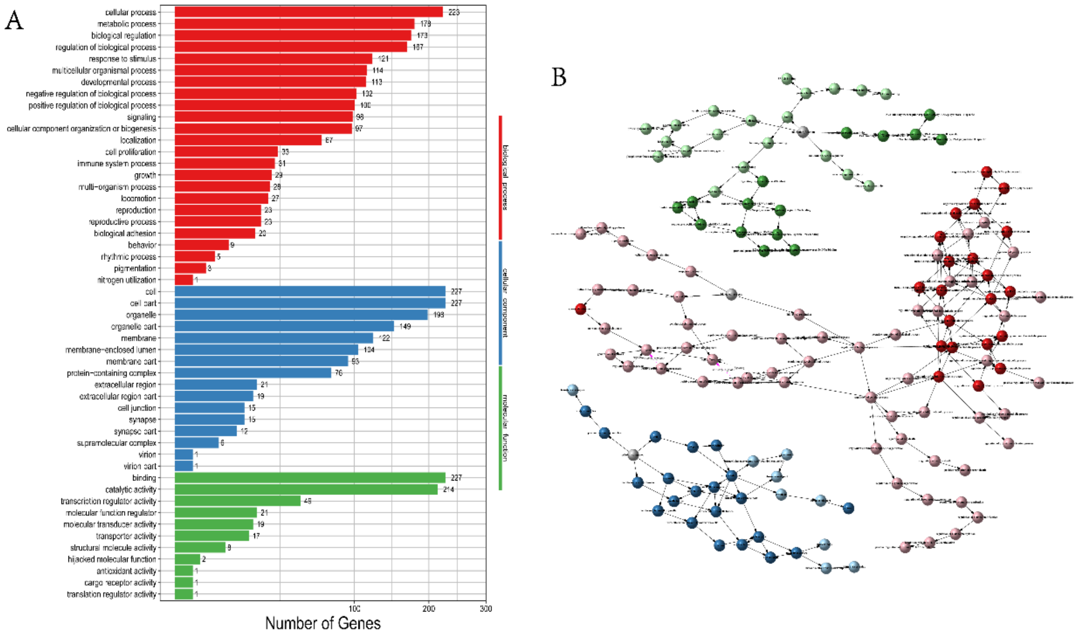

Figure 3. GO enrichment analysis of the 6 differentially expressed circRNAs. (A) The GO enrichment analysis revealed that the differentially expressed circRNAs were enriched in biological process and cellular component, molecular function. (B) Correlation network of the GO enrichment pathways. Abbreviations: GO, gene ontology.

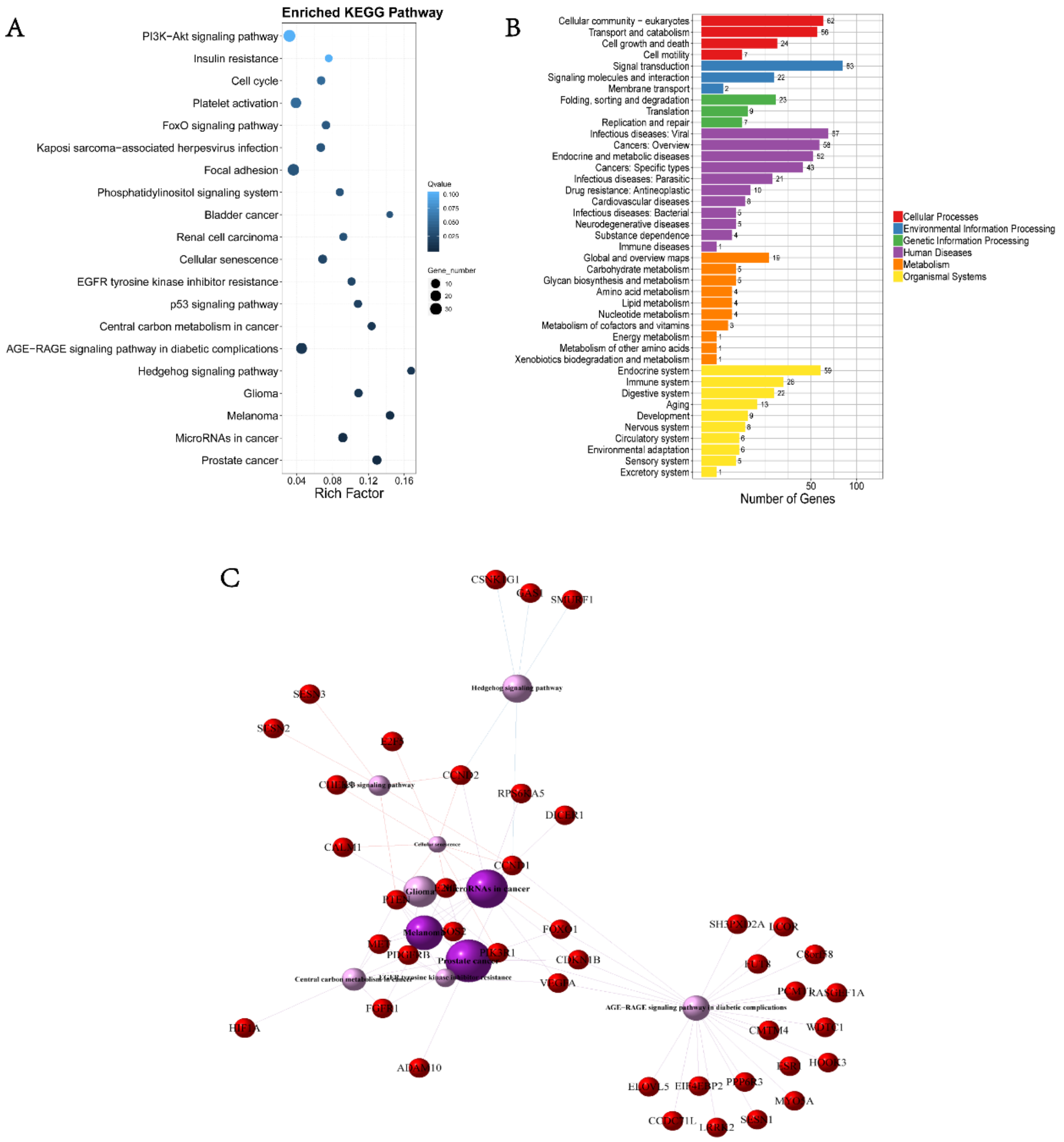

Figure 4. KEGG pathway enrichment analysis of the 6 differentially expressed circRNAs. (A) The top 20 KEGG enrichment pathways. (B) Classification of KEGG enrichment pathways. (C) Correlation network of the KEGG enrichment pathways. Abbreviations: KEGG, Kyoto Encyclopedia of Genes and Genomes.

2.Tables

Table 1. ELISA kits information

ELISA kits	Catalog numbers	Assay ranges (pg/ml)
**Aβ42**		
INNOTEST (Japan）	81576	62.5-4000
**T-tau**		
INNOTEST (Japan）	81572	34-2500
**P-tau181**		
INNOTEST (Japan)	81581	15.6-1000

Table 2. Confirmation of differential circRNAs in Dataset 2.

circRNA	log2FC (AD vs control)	*P* value
hsa_circ_0077001	0.36	< 0.001
hsa_circ_0022417	0.33	< 0.001
hsa_circ_0096551	0.29	< 0.001
hsa_circ_0020704	0.27	< 0.001
hsa_circ_0014353	0.35	< 0.001
hsa_circ_0074533	0.32	< 0.001
hsa_circ_0030167	0.27	< 0.001
hsa_circ_0077006	0.28	< 0.001
hsa_circ_0077003	0.27	< 0.001
hsa_circ_0014355	0.29	< 0.001
hsa_circ_0020706	0.30	< 0.001
hsa_circ_0020707	0.27	< 0.001
hsa_circ_0043399	0.28	< 0.001
hsa_circ_0014356	0.33	< 0.001
hsa_circ_0077002	0.28	< 0.001
hsa_circ_0051000	0.37	< 0.001
hsa_circ_0043400	0.28	< 0.001
hsa_circ_0077004	0.29	< 0.001
hsa_circ_0010881	0.29	< 0.001
hsa_circ_0051807	0.31	< 0.001
hsa_circ_0077880	0.27	< 0.001

hsa_circ_0051808	0.27	< 0.001
hsa_circ_0006940	-0.47	< 0.001
hsa_circ_0089762	-0.38	< 0.001
hsa_circ_0079274	-0.34	< 0.001
hsa_circ_0037139	-0.51	< 0.001
hsa_circ_0089761	-0.39	< 0.001
hsa_circ_0079275	-0.45	< 0.001
hsa_circ_0037140	-0.32	< 0.001
hsa_circ_0079276	-0.33	< 0.001
hsa_circ_0037141	-0.46	< 0.001
hsa_circ_0049861	-0.31	< 0.001
hsa_circ_0089894	-0.39	< 0.001
hsa_circ_0004518	-0.31	< 0.001
hsa_circ_0035110	-0.33	< 0.001
hsa_circ_0025206	-0.32	< 0.001
hsa_circ_0079268	-0.31	< 0.001
hsa_circ_0051245	-0.32	< 0.001
hsa_circ_0079272	-0.32	< 0.001
hsa_circ_0049860	-0.33	< 0.001
hsa_circ_0063372	-0.32	< 0.001

Abbreviations: CircRNA, Circular RNA; AD, Alzheimer's disease; Has, Homo sapiens; FC, fold change.

Table 3. Confirmation of differential circRNAs in Dataset 3.

**circRNA**	**log2FC (AD vs control)**	**P value**
hsa_circ_0077001	0.38	< 0.001
hsa_circ_0022417	0.37	< 0.001
hsa_circ_0096551	0.29	< 0.001
hsa_circ_0020704	0.28	< 0.001
hsa_circ_0014353	0.38	< 0.001
hsa_circ_0074533	0.37	< 0.001
hsa_circ_0030167	0.27	< 0.001
hsa_circ_0077006	0.28	< 0.001
hsa_circ_0077003	0.29	< 0.001
hsa_circ_0014355	0.28	< 0.001
hsa_circ_0020706	0.28	< 0.001
hsa_circ_0020707	0.31	< 0.001
hsa_circ_0043399	0.27	< 0.001
hsa_circ_0014356	0.39	< 0.001
hsa_circ_0077002	0.27	< 0.001
hsa_circ_0051000	0.37	< 0.001

hsa_circ_0043400	0.30	< 0.001
hsa_circ_0077004	0.29	< 0.001
hsa_circ_0010881	0.33	< 0.001
hsa_circ_0051807	0.32	< 0.001
hsa_circ_0077880	0.27	< 0.001
hsa_circ_0051808	0.34	< 0.001
hsa_circ_0006940	-0.41	< 0.001
hsa_circ_0089762	-0.39	< 0.001
hsa_circ_0079274	-0.33	< 0.001
hsa_circ_0037139	-0.37	< 0.001
hsa_circ_0089761	-0.40	< 0.001
hsa_circ_0079275	-0.39	< 0.001
hsa_circ_0037140	-0.31	< 0.001
hsa_circ_0079276	-0.33	< 0.001
hsa_circ_0037141	-0.31	< 0.001
hsa_circ_0049861	-0.32	< 0.001
hsa_circ_0089894	-0.45	< 0.001
hsa_circ_0004518	-0.28	< 0.001
hsa_circ_0035110	-0.33	< 0.001
hsa_circ_0025206	-0.29	< 0.001
hsa_circ_0079268	-0.27	< 0.001
hsa_circ_0051245	-0.27	< 0.001
hsa_circ_0079272	-0.33	< 0.001
hsa_circ_0049860	-0.34	< 0.001
hsa_circ_0063372	-0.31	< 0.001

Abbreviations: CircRNA, Circular RNA; AD, Alzheimer's disease; Has, Homo sapiens; FC, fold change.
